# Age-related differences in the characteristics of persistent postural-perceptual dizziness

**DOI:** 10.3389/fneur.2024.1378206

**Published:** 2024-04-19

**Authors:** Akina Fukushima, Kayoko Kabaya, Toshiya Minakata, Sachiyo Katsumi, Shinichi Esaki, Shinichi Iwasaki

**Affiliations:** Department of Otolaryngology, Head and Neck Surgery, Nagoya City University Graduate School of Medical Sciences, Nagoya, Japan

**Keywords:** persistent postural-perceptual dizziness, age, precipitating conditions, vestibular function, anxiety

## Abstract

**Objective:**

To investigate differences in the clinical characteristics of patients with persistent postural-perceptual dizziness (PPPD) according to age.

**Methods:**

We retrospectively reviewed 143 patients diagnosed with PPPD. Patients were classified into three groups by age: young group (19 to 44 years, *n* = 60), middle-age group (45 to 64 years, *n* = 56), old group (65 to 85 years, *n* = 27). Demographic data, scores of the Dizziness Handicap Inventory (DHI), the Niigata PPPD Questionnaire (NPQ), the Hospital Anxiety and Depression Scale (HADS), precipitating conditions, and the results of vestibular function tests including caloric testing, video head impulse test (vHIT), cervical and ocular vestibular evoked myogenic potentials (cVEMPs and oVEMPs), and posturography, were compared among the three groups.

**Results:**

While there were no significant differences in the scores of the DHI or NPQ, the total score and anxiety score in HADS in the young group were significantly higher than in the old group (*p* < 0.05, each). On the other hand, for precipitating conditions, the rate of peripheral vestibular diseases was significantly greater in the old group (77.8%) compared to the young group (41.7%, *p* < 0.01). There was no significant difference in the results of caloric testing, vHIT, cVEMPs, or oVEMPs among the three groups. For posturography, the velocity of the center of pressure with eyes-open as well as with eyes-closed was significantly greater in the old group compared to the young group and the middle-age group (*p* < 0.005, respectively).

**Conclusion:**

The clinical characteristics of PPPD were different according to age. Young patients tended to have stronger anxiety than old patients whereas the old patients had a higher proportion of peripheral vestibular diseases among the precipitating conditions compared to young patients.

## 1 Introduction

Persistent postural-perceptual dizziness (PPPD) is a functional chronic vestibular syndrome that is characterized by dizziness lasting more than 3 months exacerbated by an upright posture or walking, active or passive movement, and exposure to moving or complex visual stimuli (1). The onset of PPPD is typically preceded by peripheral and central vestibular disorders, such as vestibular neuritis, benign paroxysmal positional vertigo (BPPV), or vestibular migraine. However, other precipitants such as depression, anxiety, or central degenerative disorders may also precede (1, 2).

PPPD was originally coined as a composite of four precursor diseases: phobic postural vertigo (PPV), space-motion discomfort (SMD), visual vertigo (VV), and chronic subjective dizziness (CSD) (1). While these four diseases share clinical features that form the basis of the diagnostic criteria of PPPD, the clinical characteristics and symptoms of PPPD are heterogenous (3–5). In fact, Yagi et al. recently performed factor analysis on the clinical symptoms of PPPD patients, and classified them into the three clusters: visual-dominant, motion-dominant, and mixed subtypes (3).

The incidence and prevalence of vestibular disorders as well as mental disorders are different according to age. A population survey in Germany reported that vestibular vertigo is almost three times more frequent in the elderly compared to young adults (6). The prevalence of peripheral vestibular diseases including BPPV, vestibular neuritis, and Ménière’s disease increases with age, being highest at over 70 years old (7). On the other hand, the onset of the most mental disorders is below 25 years old (8). The prevalence rates are highest at 15–25 years for anxiety disorders and at 35–49 years old for depression, and both anxiety and depression decrease in the elderly (more than 65 years old) (9–11).

While the average age of PPPD patients is in the mid-40s, ranging from adolescence to late adulthood (1), it is possible that the clinical characteristics of PPPD differ according to the patients’ age. The objective of this study was to examine whether there are any age-related differences in the clinical characteristics of PPPD patients including vestibular and psychological symptoms, precipitating conditions, and vestibular function.

## 2 Materials and methods

This study was a retrospective chart review in a tertiary referral center. This study was approved by the Research Ethics Committee, Graduate School of Medicine, Nagoya City University (60-22-0106) and was conducted according to the tenets of the Declaration of Helsinki.

### 2.1 Subjects

We reviewed the clinical records of 143 patients who were newly-diagnosed as having PPPD at the Department of Otolaryngology, Head and Neck Surgery, Nagoya City University Hospital from January 2019 to December 2022. We diagnosed PPPD according to the diagnostic criteria of the Bárány Society (1). All of the patients received detailed medical interviews and physical examinations, a neurological examination, pure-tone audiometry, and positional/positioning nystagmus testing under infrared CCD goggles. In addition, they answered three questionnaires at their initial visit: the Dizziness Handicap Inventory (DHI) (12), the Niigata PPPD Questionnaire (NPQ) (13), and the Hospital Anxiety and Depression Scale (HADS) (14). They underwent vestibular function testing including caloric testing, the video head impulse test (vHIT), cervical and ocular vestibular evoked myogenic potentials (cVEMPs and oVEMPs), and posturography.

#### 2.1.1 Precipitating conditions

All patients were identified precipitating conditions before the onset of PPPD. The method of identification of precipitating conditions was based on the medical records of the referring physician and/or our clinic and a detailed medical interview with the patients, with additional physical examinations if necessary. The diagnostic criteria used in this study were: BPPV (15), Ménière’s disease (16), vestibular neuritis (17), sudden deafness with vertigo (18), delayed endolymphatic hydrops (19), Ramsay Hunt syndrome (20), vestibular migraine (21), psychiatric dizziness; vestibular symptom due to panic disorder, anxiety, or depression (22), orthostatic dysregulation (23). We defined peripheral vestibular dysfunction as comprising patients who experienced vertigo or dizziness, showing abnormalities in one or more vestibular function tests, such as caloric testing, vHIT, cVEMPs, or oVEMPs, yet did not satisfy the established criteria for peripheral vestibular diseases (BPPV, Ménière’s disease, vestibular neuritis etc.). Definitions of abnormalities are given in the vestibular function tests section below.

#### 2.1.2 Questionnaires

The DHI is a 25-item self-assessment scale designed to evaluate the self-perceived handicap caused by dizziness (12). The DHI includes three subscales: functional (9 items), emotional (9 items), and physical (7 items). Each item can be scored as a 4 (yes), 2 (sometimes), or 0 (no). A DHI score of 0 means no handicap, and 100 means a significant perceived handicap due to dizziness.

The NPQ was developed for making diagnoses as well as for assessing the severity of PPPD (13). The NPQ consists of 12 questions that evaluate the degree of symptom exacerbation by three exacerbating factors: upright posture or walking, active or passive movement, and visual stimulation. The severity of each factor is evaluated using 4 questions which are scored from 0 (none) to 6 (unbearable).

The HADS is a 14-item self-assessment questionnaire consisting of 7 items each on anxiety and depression (14). Each question is scored on a four-point scale (0–3). The total score ranges from 0 (no anxiety and depression) to 21 (high anxiety and strong depression).

#### 2.1.3 Vestibular function tests

Caloric testing was carried out using air at 24°C and 50°C for 60 s each. Maximum slow-phase eye velocity (MSPV) was measured using video-nystagmography and canal paresis (CP)% was calculated using Jongkee’s index formula (24). An abnormal caloric response was defined as having either of the following criteria: (i) CP% greater than 20% for unilateral dysfunction (25) or (ii) MSPV <10 deg./s bilaterally for bilateral dysfunction (26).

The vHIT was performed to assess the vestibulo-ocular reflex (VOR) in the three semicircular canal planes using an Eye-See-Cam system (Interacoustics, Denmark). The vHIT was used to measure VOR gain, the ratio of the eye velocity to head velocity when the head is rotated quickly through an angle of 10–20° with the eyes fixed on a target. In this study, a mean VOR gain <0.8 for the horizontal semicircular canal and <0.7 for the vertical canals was considered abnormal (27).

The testing of cVEMPs assessed the saccule-inferior vestibular nerve function and the testing of oVEMPs assessed the utricle-superior vestibular nerve function, using the Neuropack system (Nihon Koden, Japan). Short-tone bursts of 500 Hz (95 dB normal hearing level, 135 dB SPL (peak value), rise/fall time = 1 ms, plateau time = 2 ms) were used with air conduction for cVEMPs and oVEMPs. The amplitude of p13-n23 was recorded at the sternocleidomastoid muscles (SCM) in cVEMPs and the amplitude of n10-p15 was recorded at external eye muscles in oVEMPs. We calculated the asymmetry ratio (AR) for the amplitude with the following formula using the amplitude on the affected side (Aa) and that on the unaffected side (Au): AR (%) = 100 * (Au − Aa)/(Au + Aa). On the basis of results from normal subjects, the upper limit of the AR was set to 34.0 for cVEMPs (28) and 34.4 for oVEMPs (29). When no reproducible waveforms were present in 2 consecutive runs, we regarded it as an “absent” response. When a reproducible waveform was present and the AR was greater than the predefined upper limit for normal subjects, we regarded it as a “decreased” response. Both “decreased” and “absent” responses were classified as abnormal.

Posturography was used to assess the sway of the center of pressure (COP) in a standing posture, using a Gravicorder GW-5000 (Anima, Japan). The sway path with eyes-open and eyes-closed in a standing posture was measured for 60 s. We assessed the velocity of the movement of the COP with eyes-open and eyes-closed and the Romberg ratio of velocity.

### 2.2 Statistical analysis

The Shapiro–Wilk test was used to check the normal distribution of the data. The Kruskal–Wallis test was used to compare continuous data. The Bonferroni test was used as a post-hoc test and the Mann–Whitney U test was used for pairwise comparisons. We used Fisher’s exact test to evaluate binary data. All statistical tests were two-sided. A difference of *p* < 0.05 was considered significant. All statistical analyses were performed using EZR version 1.37 for Windows (Saitama Medical Center, Jichi Medical University, Saitama, Japan) (30).

## 3 Results

We classified 143 PPPD patients (mean age ± SD: 49.2 ± 15 years [range, 19–85]; 40 men and 103 women) into the three groups according their age: (1) the young group (age range: 19–44 years; *n* = 60), (2) the middle-age group (age range: 45–64 years; *n* = 56) and (3) the old group (age range: 65–85 years; *n* = 28). Table 1 shows the demographics of these patients. There were no significant differences in the gender or the duration from the onset of symptoms among the three groups (*p* = 0.831 for gender and *p* = 0.069 for duration from onset).

**Table 1:** Demographics of PPPD patients grouped by age.

	Young (19–44 years) *N* = 60	Middle-age (45–64 years) *N* = 56	Old (65–85 years) *N* = 27	*p*-value
Age: mean (SD) years	35.0 (7.0)	53.6 (5.5)	71.5 (5.6)	<0.001
Gender, *N* (%)				
Male	15 (25.0%)	17 (30.4%)	8 (29.6%)	0.831
Female	45 (75.0%)	39 (69.6%)	19 (70.4%)	
Duration: mean (SD) months	25.0 (34.7)	39.0 (45.4)	44.8 (48.7)	0.069

SD, standard deviation; Duration, duration from symptom onset.

**Figure 1:**
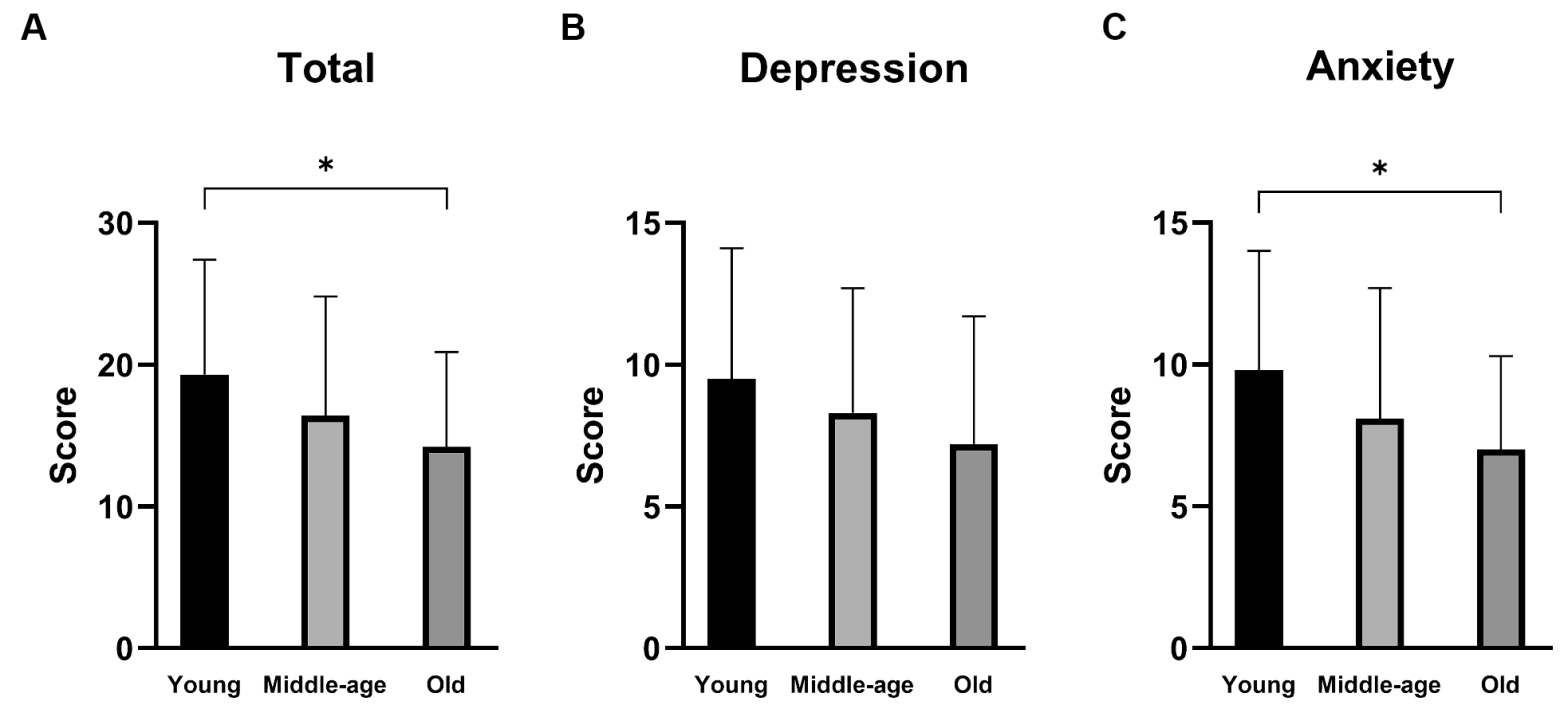
Hospital Anxiety and Depression Scale (HADS) scores of the PPPD patients in different age groups. **(A)** The total scores of HADS. The total scores were significantly different among the three groups (*p* = 0.022). The young group showed a significantly higher score than the old group (*p* = 0.029). **(B)** The depression subscale scores of HADS. There was no significant difference among the three groups. **(C)** The anxiety subscale of HADS. The anxiety subscale scores were significantly different among the three groups (*p* = 0.013). The young group showed a significantly higher score than the old group (*p* = 0.022). ^*^p < 0.05.

The scores of the DHI or NPQ were not significantly different among the three groups (one-way ANOVA: *p* > 0.05 each). On the other hand, there were significant differences in the total scores as well as the anxiety subscales of HADS among the three groups (one-way ANOVA: *p* = 0.022 for total score and *p* = 0.013 for anxiety subscale; Table 2 and Figure 1). *Post hoc*-analysis revealed that both the total scores and anxiety subscales of HADS in the young group were significantly higher than those of the old group (Bonferroni test: *p* = 0.029 for total score and *p* = 0.022 for anxiety subscale).

**Table 2:** DHI, NPQ, and HADS of PPPD patients grouped by age.

	Young (19–44 years) *N* = 60	Middle-age (45–64 years) *N* = 56	Old (65–85 years) *N* = 27	*p*-value
DHI				
Total	60.9 (21.0)	59.4 (20.0)	55.4 (22.5)	0.542
Physical	17.3 (5.9)	18.5 (6.4)	16.2 (6.9)	0.365
Emotional	22.1 (7.5)	19.5 (8.4)	18.9 (8.8)	0.142
Functional	21.5 (9.8)	21.4 (8.9)	19.0 (10.2)	0.549
NPQ				
Total	41.1 (14.8)	40.9 (13.0)	37.0 (12.1)	0.451
Standing	13.9 (6.3)	12.5 (5.2)	12.7 (4.6)	0.281
Movement	13.4 (5.0)	13.3 (4.8)	11.5 (4.8)	0.245
Visual	13.8 (5.8)	15.0 (4.8)	12.8 (5.4)	0.243
HADS				
Total	19.3 (8.1)	16.4 (8.4)	14.2 (6.7)	0.022^*^
Depression	9.5 (4.6)	8.3 (4.4)	7.2 (4.5)	0.057
Anxiety	9.8 (4.2)	8.1 (4.6)	7.0 (3.3)	0.013^*^

Data are shown as mean (SD). DHI, dizziness handicap inventory; NPQ, the Niigata PPPD questionnaire; HADS, hospital anxiety and depression scale. ^*^p < 0.05.

Table 3 shows the precipitating conditions in each PPPD patient group. In all the three groups, BPPV and Ménière’s disease were the most common. Peripheral vestibular diseases accounted for 41.7% of the precipitating conditions in the young group, 55.4% in the middle-age group, and 77.8% in the old group. There were significant differences among them (Fisher’s exact test, *p* = 0.007), and the rate of peripheral vestibular diseases in the old group was significantly greater than that in the young group (Bonferroni test: *p* = 0.007). While vestibular migraine was common in the young and middle-age groups (11.7 and 12.5%, respectively), there were no patients with this disorder in the old group (Fisher’s exact test, *p* = 0.134). Psychiatric dizziness was more frequent in the middle-age group (10.7%) compared to the young or old groups (5 and 3.7%, respectively; Fisher’s exact test, *p* = 0.468).

**Table 3:** Precipitating conditions of PPPD patients grouped by age.

	Young (19–44 years) *N* = 60	Middle-age (45–64 years) *N* = 56	Old (65–85 years) *N* = 27
Peripheral vestibular diseases	25 (41.7%)	31 (55.4%)	21 (77.8%)
Benign paroxysmal positional vertigo	9 (15.0%)	9 (16.1%)	7 (25.9%)
Ménière’s disease	8 (13.3%)	10 (17.9%)	6 (22.2%)
Vestibular neuritis	1 (1.7%)	2 (3.6%)	2 (7.4%)
Sudden deafness with vertigo		2 (3.6%)	2 (7.4%)
Delayed endolymphatic hydrops		1 (1.8%)	
Ramsay-Hunt syndrome		1 (1.8%)	1 (3.7%)
Peripheral vestibular dysfunction	7 (11.7%)	6 (10.7%)	3 (11.1%)
Other conditions	35 (58.3%)	25 (44.6%)	6 (22.2%)
Vestibular migraine	7 (11.7%)	7 (12.5%)	
Psychiatric dizziness	3 (5.0%)	6 (10.7%)	1 (3.7%)
Orthostatic dysregulation	1 (1.7%)	1 (1.8%)	
Dizziness of unknown cause	19 (31.7%)	5 (8.9%)	4 (14.8%)
No precipitating conditions	5 (8.3%)	6 (10.7%)	1 (3.7%)

Data are shown as number (%).

**Figure 2:**
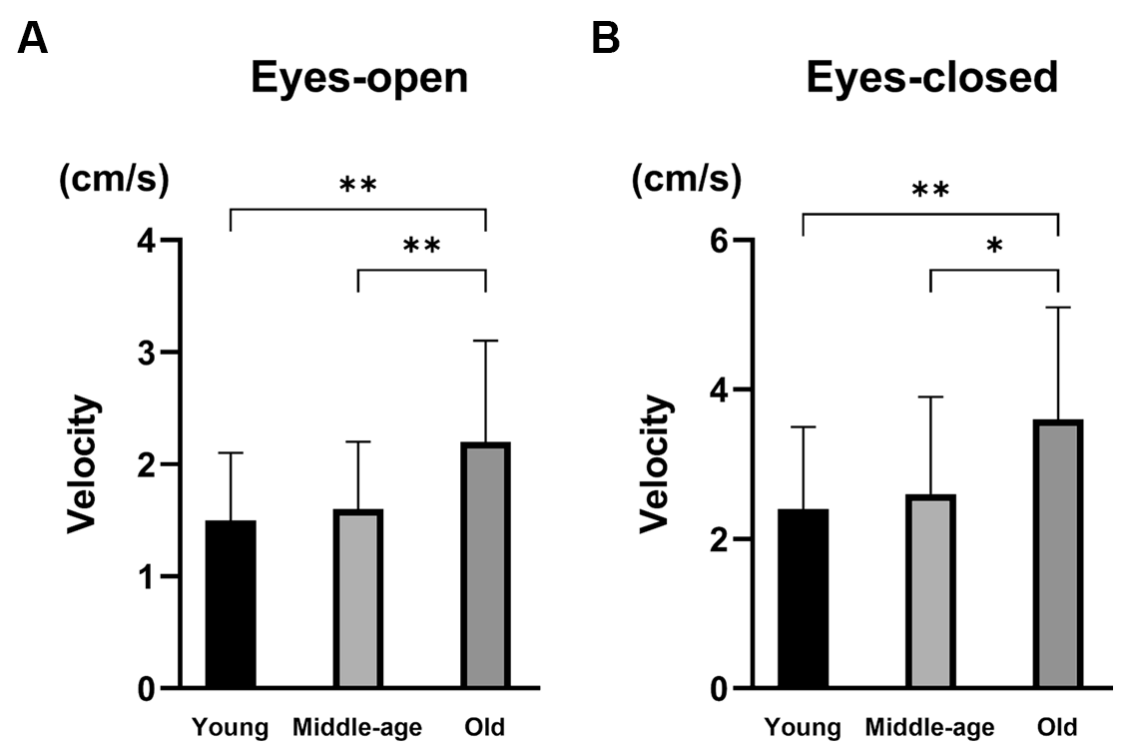
The results of posturography in PPPD patients with different ages. **(A)** The velocity of the center of pressure (COP) with eyes-open. The velocities of the COP with eyes-open were significantly larger in the old group than in the young group (*p* = 0.001) and the middle-age group (*p* = 0.007). **(B)** The velocity of COP with eyes-closed. The velocities of the COP with eyes-closed were significantly larger in the old group than in the young group (*p* = 0.002) and the middle-age group (*p* = 0.014). ^*^p < 0.05 and ^**^p < 0.01.

Table 4 shows the results of vestibular function tests in the three PPPD patient groups. There were no significant differences in the abnormality ratios in the caloric test, vHIT, cVEMPs, or oVEMPs among the three groups (Fisher’s exact test, *p* = 0.328, *p* = 0.188, *p* = 0.074, and *p* = 0.767, respectively). For posturography, the velocities of the COP with eyes-open and eyes-closed in the old group were significantly greater than those in the young group and the middle-age group (Kruskal–Wallis test, *p* = 0.001 and *p* = 0.002, respectively; Figure 2). There were no significant differences in the Romberg ratios of the velocity of the COP among the three groups (Kruskal–Wallis test, *p* = 0.335).

**Table 4:** Results of vestibular function tests in PPPD patients grouped by age.

		Young (19–44 years)	Middle-age (45–64 years)	Old (65–85 years)	*p*-value
Caloric testing	Normal	30 (68.2%)	28 (54.9%)	10 (53.6%)	0.328
	Abnormal	14 (31.8%)	23 (45.1%)	9 (47.4%)	
vHIT	Normal	33 (94.3%)	29 (85.3%)	7 (77.8%)	0.188
	Abnormal	2 (5.7%)	5 (14.7%)	2 (22.2%)	
cVEMPs	Normal	26 (83.9%)	23 (71.9%)	4 (44.4%)	0.074
	Abnormal	5 (16.1%)	9 (28.1%)	5 (55.6%)	
oVEMPs	Normal	12 (63.2%)	13 (52.0%)	2 (40.0%)	0.767
	Abnormal	7 (36.8%)	12 (48.0%)	3 (60.0%)	
Posturography					
Romberg ratio of velocity		1.6 (0.5)	1.6 (0.4)	1.7 (0.5)	0.335
Velocity with eyes-open	(cm/s)	1.5 (0.6)	1.6 (0.6)	2.2 (0.9)	0.001
Velocity with eyes-closed	(cm/s)	2.4 (1.1)	2.6 (1.3)	3.6 (1.5)	0.002

Data are shown as number (%) in caloric testing, vHIT, cVEMPs, and oVEMPs. Data are shown as mean (SD) in Romberg ratio and velocity of posturograhy.

## 4 Discussion

In the present study, we investigated the differences in the clinical characteristics of PPPD patients classified according to their age, and revealed that the total HADS score and its anxiety subscale were significantly higher in the young group compared to the old group while there were no differences in the DHI or NPQ scores between them. On the other hand, the proportion of peripheral vestibular diseases among the precipitating conditions was significantly higher in the old group compared to the young and middle-age groups. The abnormal ratios of the vestibular function tests were not significantly different among the three age groups. Our results suggest that the clinical characteristics of PPPD patients in different age groups differ in their psychiatric conditions as well as in the precipitating conditions.

The diagnostic criteria of PPPD were made based on the common clinical features of the four precursors: PPV, SMD, VV, and CSD. However, it is still under debate whether PPPD is a single disorder with one pathophysiologic mechanism or just a composite of multiple conditions that produce similar symptoms from different mechanisms (1). PPV, SMD, VV, and CSD originally have different areas of emphasis: postural provocation is the characteristic feature of PPV, discomfort in self-motion is emphasized in SMD and CSD, and trouble with moving visual stimuli is the primary feature of VV. Thus, the clinical characteristics of PPPD are heterogeneous, and several subtypes may exist. Yagi et al. performed factor and cluster analyses on answers to the NPQ in PPPD patients, and revealed that PPPD patients can be categorized into three clusters: the visual-dominant subtype, the motion-dominant subtype, and the mixed subtype (3). In the present study, there were no significant differences in the scores of standing, motion, or visual subscales of the NPQ among the different age groups of PPPD patients.

The pathophysiological mechanisms of PPPD are still unclear. Anxiety-related personality traits and high levels of anxiety and vigilance about acute vestibular symptoms have been associated with the initial pathologic processes (1). Alterations in postural control strategies (31), shifts in multi-sensory information (32), and reduced cortical integration of spatial orientation (33) have been considered as the underlying mechanisms of sustained dizziness in PPPD. Previous studies have shown that patients with a history of anxiety disorders before the onset of vestibular symptoms were more at risk of developing chronic dizziness (34, 35). In contrast, patients with higher resilience, sense of coherence and general satisfaction with life were less likely to develop persistent dizziness after an acute vestibular event (36). In the present study, the total score of HADS and its anxiety subscale in the young group were significantly higher compared to those in the old group, suggesting that psychological factors, especially the degree of anxiety, are more severe in the younger PPPD patients than in older patients. This result might have an association with the early onset of mental disorders. Epidemiological studies have shown that more than half of the patients with any mental disorders including anxiety disorders develop symptoms at under 25 years old (8) and that the prevalence rate of anxiety disorders is highest at 15–25 years (9, 10). It is also possible that vestibular symptoms in younger patients can lead to psychiatric comorbidities (37, 38). It has been reported that distress due to dizziness was more severe in younger patients with chronic dizziness in comparison with older patients (37) and that the tolerance to dizziness intensity increases with age in chronic dizziness (38). Our result is compatible with a previous study that compared the clinical characteristics of PPPD patients in different age groups (39). It reported that the proportion of PPPD patients having emotional disorders and the scores of the Beck anxiety inventory were lower in the old group than in the middle-age group (39).

PPPD is usually precipitated by conditions that cause vertigo, unsteadiness or balance problems (1). Previous studies reported that the most common precipitating conditions are peripheral vestibular diseases including BPPV, vestibular neuritis and Ménière’s disease (1, 3, 39). In contrast, another study reported that psychological distress in the form of post-traumatic stress disorder was the most frequent co-morbidity followed by vestibular migraine (40). These studies suggest that the nature of the precipitating conditions may vary depending on clinical departments, institutions, or countries. In the present study, the rate of peripheral vestibular diseases in the precipitating conditions was significantly higher in the old group than in the young group of PPPD patients. Our results might have an association with a higher incidence of peripheral vestibular diseases including BPPV, vestibular neuritis and Ménière’s disease in the elderly (6, 7, 41). On the other hand, psychiatric dizziness exhibited a greater prevalence in the middle-age group compared to the young or old groups among the precipitating conditions. This finding was consistent with a previous study demonstrating a higher prevalence of psychiatric comorbidities among middle-aged PPPD patients compared to their younger or older counterparts (39).

In the present study, there were no significant differences in the abnormal ratios of the vestibular function tests including caloric testing, vHIT, cVEMPs or oVEMPs among the three different age groups of PPPD patients. A previous study examined the association between vestibular function and preceding balance disorders in PPPD patient (42). It showed that PPPD patients with preceding vestibular neuritis had a significant positive association with abnormal caloric responses whereas patients with preceding BPPV had significantly lower rates of abnormal oVEMPs. While more than half of PPPD patients exhibited peripheral vestibular diseases as precipitating conditions in the present study, the rate of abnormalities in the vestibular function test was relatively diminished. These may be due to the considerable intervals between the onset of precipitating conditions and the administration of vestibular function assessments, along with the elevated prevalence of BPPV among the precipitating conditions. In posturography, the velocity of the COP with eyes-open as well as with eyes-closed was significantly greater in the old group than in the young group in the present study. This might be due to deterioration of postural stability with aging (43).

This study has several limitations. First, this is a retrospective study. There is the potential risk for selection bias in the distribution of patients with PPPD. Second, the number of patients in each group was small. Third, we could not examine the association between the effect of treatment and ages of the patients since the treatment strategies varied among the patients. A large-scale study including multiple clinics taking into the long-term outcome is necessary to elucidate the characteristics of PPPD with different ages.

In conclusion, we investigated the differences in the clinical characteristics of PPPD patients classified according to age, and showed that younger patients tended to have stronger anxiety than older patients whereas the older patients had a higher proportion of peripheral vestibular diseases in the precipitating conditions compared to the younger patients. Our results suggest that in treating PPPD patients, psychiatric conditions such as anxiety and depression should be carefully checked in younger patients whereas peripheral vestibular comorbidities should be taken into account when treating older PPPD patients.

## Data availability statement

The raw data supporting the conclusions of this article will be made available by the authors, without undue reservation.

## Ethics statement

The studies involving humans were approved by the Research Ethics Committee, Graduate School of Medicine, Nagoya City University. The studies were conducted in accordance with the local legislation and institutional requirements. The participants provided their written informed consent to participate in this study.

## Author contributions

AF: Writing – review & editing, Writing – original draft. KK: Writing – review & editing, Writing – original draft. TM: Writing – review & editing. SK: Writing – review & editing. SE: Writing – review & editing. SI: Writing – review & editing, Writing – original draft.
